# Molecular and Structural Evolution of Porcine Epidemic Diarrhea Virus

**DOI:** 10.3390/ani12233388

**Published:** 2022-12-01

**Authors:** Baicheng Huang, Guoqian Gu, Yunjing Zhang, Zhenzhen Chen, Kegong Tian

**Affiliations:** 1 National Research Center for Veterinary Medicine, Luoyang 471000, China; 2 Research Center for Intelligent Computing Platforms, Zhejiang Laboratory, Hangzhou 311100, China; 3 Department of Infectious Diseases and Public Health, Jockey Club College of Veterinary Medicine, City University of Hong Kong, Hong Kong SAR 999077, China; 4 College of Veterinary Medicine, Henan Agricultural University, Zhengzhou 450046, China

**Keywords:** porcine epidemic diarrhea virus, molecular evolution, positive selection, structural assay, haplotypes

## Abstract

**Simple Summary:**

To analyze the evolutionary characteristics of the highly contagious porcine epidemic diarrhea virus (PEDV), the complete genomes of 647 PEDV strains were analyzed. Eight amino acid (aa) sites of the S protein showed strong signals of positive selection, and seven of them were located on the surface of the S protein (S1 domain), suggesting a high selection pressure of S protein. Topologically, the S gene is more representative of the evolutionary relationship at the genome-wide level than are other genes. Structurally, the evolutionary pattern is highly S1 domain-related. The haplotype networks of the S gene showed that the strains are obviously clustered geographically in the lineages corresponding to genotypes GI and GII. The three distinguishable nucleic acid sites in the haplotypes assay suggested a putative evolutionary mechanism in PEDV. These findings provide several new fundamental insights into the evolution of PEDV and guidance for developing effective prevention countermeasures against PEDV.

**Abstract:**

To analyze the evolutionary characteristics of the highly contagious porcine epidemic diarrhea virus (PEDV) at the molecular and structural levels, we analyzed the complete genomes of 647 strains retrieved from the GenBank database. The results showed that the spike (S) gene exhibited larger dS (synonymous substitutions per synonymous site) values than other PEDV genes. In the selective pressure analysis, eight amino acid (aa) sites of the S protein showed strong signals of positive selection, and seven of them were located on the surface of the S protein (S1 domain), suggesting a high selection pressure of S protein. Topologically, the S gene is more representative of the evolutionary relationship at the genome-wide level than are other genes. Structurally, the evolutionary pattern is highly S1 domain-related. The haplotype networks of the S gene showed that the strains are obviously clustered geographically in the lineages corresponding to genotypes GI and GII. The alignment analysis on representative strains of the main haplotypes revealed three distinguishable nucleic acid sites among those strains, suggesting a putative evolutionary mechanism in PEDV. These findings provide several new fundamental insights into the evolution of PEDV and guidance for developing effective prevention countermeasures against PEDV.

## 1. Introduction

The enteric disease porcine epidemic diarrhea (PED) in suckling piglets, characterized by severe diarrhea, vomiting and dehydration, was first reported in Europe in the early 1970s [1]. The etiology of porcine epidemic diarrhea virus (PEDV) has caused severe epidemics since the 1990s in Asia, including Japan [2] and Korea [3]. The devastating outbreaks of PEDV in China since 2006 [4,5,6], and in the United States since 2013 [7,8] led to a serious threat to swine health. Until now, PEDV has been the primary viral pathogen causing porcine diarrhea in China. The mortality rate of suckling piglets caused by PED often reaches 80–100%, resulting in serious economic losses in the swine industry.

PEDV is an enveloped, positive-sense, single-stranded RNA virus, belonging to the genus *Alphacoronavirus* in the family Coronaviridae of the order Nidovirales. Among the four PEDV structural proteins, namely spike (S), envelope (E), membrane (M), and nucleocapsid (N), the S protein dominates the surface of virus particles and mediates direct binding to cell receptors, is a major target to induce neutralizing antibodies [9,10,11], and can also be the target in vaccine development [12]. In coronavirus, as the major surface protein that directly interacts with cellular receptors, the S protein bears the greatest evolutionary pressure and the S gene contains the most variable regions in the entire PEDV genome, including the strains reported in the United States in 2013–2014 [8,13] with insertions and deletions in the S gene (S-INDEL). Thus, the diversity of the S gene is the phylogenetic marker in PEDV evolutionary analysis [13,14,15].

Despite the discoveries in the levels of the complete genome and the S gene, several fundamental issues related to the evolutionary patterns of PEDV remain unknown. In this study, we investigated the molecular divergence between PEDV and other related coronaviruses and carried out genetic analyses of PEDV complete genomes and structural protein genes, particularly from the viewpoint of the crystal structure of the S protein. This work could provide new insights into the evolution of PEDV and its spread pattern in animals.

## 2. Materials and Methods

### 2.1. Data Collection of PEDV and Other Related Viruses

To analyze the viral evolution of PEDV, part of the PEDV complete genome sequences in GenBank (https://www.ncbi.nlm.nih.gov/genbank, accessed on 20 June 2022) were not analyzed due to the high cell-adapted passages, or the artificially modified mutations. So, a total of 647 qualified whole-genome sequences of PEDV (from 22 countries) were selected for analysis in this study. Genome sequences of other related viruses were downloaded from GenBank. Details on the data set are summarized in Supplementary Table S1 and Figure S1.

### 2.2. Phylogenetic Analyses

The sequences of the PEDV complete genome, S gene and the combined gene fragments (ORF3, E, M, and N) were aligned using MUSCLE v3.8 [16]. The alignments were trimmed and edited by trimAl [17]. The codon alignments of the conserved ORFs of ORF1ab, S, ORF3, E, M, and N were further concatenated for down-stream evolutionary analysis. To infer phylogenetic trees, an ML approach was applied using RAxML [18]. The best-fit nucleotide substitution model was determined using Module Test-NG [19]. The model GTR + I + G4 was used in RAxML, which was run on 1000 bootstrap replicates.

### 2.3. Positive Selection of Amino Acids

The ratio of dN (nonsynonymous substitutions per nonsynonymous site) to dS (synonymous substitutions per synonymous site) substitution rates was identified as a value of ω (dN/dS). Positive selection was analyzed using EasyCodeML [20]. The M7 (beta) and M8 (beta and ω > 1) models were compared. In the M7 model, ω followed a beta distribution (0 ≤ ω ≤ 1), and in the M8 model, a proportion p0 of sites had ω drawn from the beta distribution, and the remaining sites with proportion p1 were positively selected and had ω1 > 1 [21]. After a likelihood-ratio test (LRT) for the pairwise comparisons of codon models using EasyCodeML, the naive empirical Bayes (NEB) and Bayes empirical Bayes (BEB) methods [22] were used to identify amino acid residues that potentially evolved under selection. The threshold for identifying amino acid sites under selection is a posterior probability of 0.95 [23].

### 2.4. Codon Usage Bias Analysis

The RSCU (Relative Synonymous Codon Usage) values of each codon in the PEDV CV777 genome (AF353511.1) were calculated. The RSCU value for each codon was the observed frequency of this codon divided by its expected frequency under equal usage among the amino acid [24]. The codons with RSCU > 1 were defined as preferred codons, and those with RSCU < 1 were defined as unpreferred codons. The FOP (frequency of optimal codons) value of each gene was calculated as the number of preferred codons divided by the total number of preferred and unpreferred codons.

### 2.5. Amino Acid Alignment

Amino acid sequences of S and N proteins were aligned to reveal the sequence identity using ESPript 3.0 [25]. The positively selected sites in the S and N proteins from NEB analysis were labeled (*: *p* > 95%; **: *p* > 99%).

### 2.6. Protein Spatial Structure Analysis

We generated aligned sequences of S protein to reveal the highly mutated regions using the sequence logo generator WebLogo 3 [26]. The highly mutated regions in the S protein were labeled in the structural model (spheres mode) of the S protein based on the PDB 6vv5 [27]; the brown transparent labeled region indicated the S1 domain.

### 2.7. Haplotype Network

The software DnaSP v6 [28] was used to generate multi-sequence aligned haplotype data, and PopART v1.7 [29] was used to draw haplotype networks based on the haplotypes generated by DnaSP v6. The evolution pattern of the PEDV was analyzed based on the representative strains identified by dominant haplotypes using MEGA-X [30].

## 3. Results

### 3.1. The Divergence between PEDV and Other Related Coronaviruses

We concatenated six ORFs (ORF1ab, S, ORF3, E, M, N) and used CODEML in the PAMLX to calculate the pairwise dN, dS, and ω values between PEDV and other viruses (Table 1). The results showed that the dS value varied across genes in CV777 and the other viruses, and that the S gene exhibited larger dS values than did other genes (Table 1), which could be caused by a high mutation rate or by natural selection that favors synonymous substitutions. The results of codon usage bias analysis (Supplementary Table S2) showed no difference between the frequency of optimal codons (FOP) of the S gene and the genomic average (0.656 versus 0.659).

### 3.2. Positive Selection of PEDV and Related Coronaviruses

**Figure 1:**
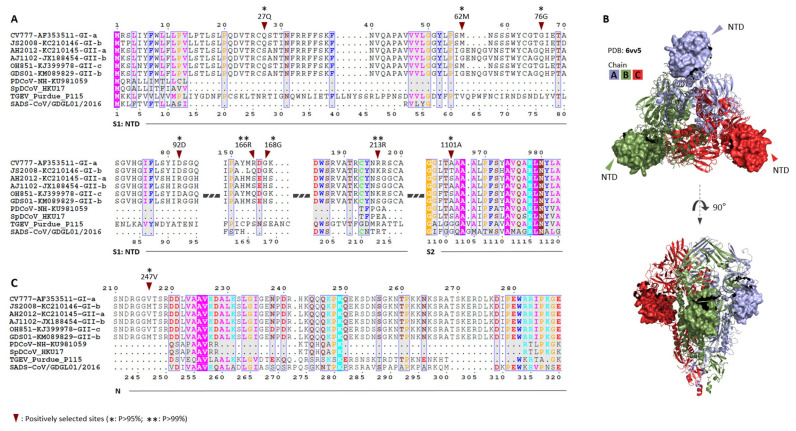
Selective pressures during the evolution of PEDV and related viruses: (**A**) eight positively selected sites in the spike (S) protein by NEB analysis, seven of them in the domain NTD, and one in the domain S2 (*: *p* > 95%; **: *p* > 99%); (**B**) the NTD domain in the surface of the S protein, showed as a surface model based on the PDB file 6vv5; (**C**) one positively selected site in the N protein by NEB analysis (*: *p* > 95%).

The genome-wide *ω* value from 0.0782 to 0.3278 (Table 1) between PEDV CV777 and other viruses indicates a strong negative selection on the nonsynonymous sites, which means that from 67.22% to 92.18% of the nonsynonymous mutations were removed during viral evolution. In the assay of extent of positive selection, the S genes of all the viruses were analyzed using the M7 (beta: neutral and negative selection) and M8 (beta & *ω* > 1: neutral, negative, and positive selection) models in CODEML. The M8 model (lnL = −18,515.815, np = 22) was a significantly better fit than the M7 (lnL = −18,531.752, np = 20) model (*p* = 1.20 × 10^−7^), suggesting that some aa substitutions were favored by positive selection. Under the M8 model, 91.436% (p0) of the nonsynonymous substitutions were estimated under neutral evolution or purifying selection (0 ≤ ω ≤1), and 8.564% (p1) of the nonsynonymous substitutions were under positive selection (*ω* = 1.503). A naive empirical Bayes (NEB) analysis suggested that eight aa sites showed strong signals of positive selection, seven of these positively selected sites were in the N-terminal domain (NTD) of the S protein (surface of the S protein), and one site (1101A) was in the S2 domain (Figure 1A,B, Supplementary Figures S2 and S3). Two sites (166R and 213R) of the S protein with the highest values of “post mean +- SE for *ω*” in the NEB analysis were also identified as positively selected sites in the Bayes empirical Bayes (BEB) analysis. The results of the positive selection assay indicated that these sites were responsible for the evolution of S protein sequences, and deserve further functional studies. In the N gene, the M8 model (lnL = −5144.209, np = 22) was not a better fit than the M7 (lnL = −5145.170, np = 20) model (*p* = 0.382), suggesting that positive selection was not favored. Only one aa site on the N protein showed a strong signal for positive selection (247V) in the NEB analysis (Figure 1C), but it did not in the BEB analysis.

### 3.3. Molecular Phylogenetic Analysis of PEDV Strains

**Figure 2:**
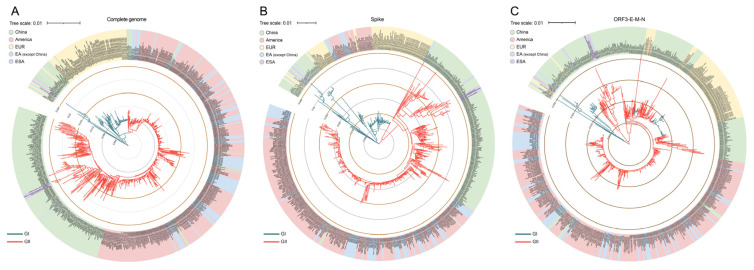
Genotyping and origin of the 647 PEDV strains based on different genes. Phylogenetic trees were constructed by the maximum-likelihood (ML) method based on: (**A**) complete genome, (**B**) S gene, and (**C**) ORF3-E-M-N gene sequences, with 1000 bootstrap replicates. Names of strains, isolation regions (EUR, Europe; EA, Japan and South Korean; ESA, Southeast Asia), GenBank accession numbers and genogroups are shown.

The phylogenetic trees of PEDVs were constructed based on the sequence of the complete genome (represented by six concatenated ORFs, including ORF1ab, S, ORF3, E, M and N), spike, and ORF3-E-M-N, respectively. Topologically, as shown in Figure 2, the similarity between the complete genome and spike is higher than that of the complete genome and ORF3-E-M-N. Genotypes of G1 and G2 showed clear differentiation in both the phylogenetic trees of the complete genome and spike, while the tree of ORF3-E-M-N was cross-connected topologically compared with the complete genome and spike, indicating that the spike gene is more representative of the evolutionary relationship at the genome-wide level than are other genes. Geographically, the American strains in genotype GI were clustered with most of the strains from Japan and South Korea. The strains from China were distributed in both the genotypes GI and GII with a similar ratio in all three phylogenetic trees.

### 3.4. PEDV Mutation from the Perspective of S Protein Spatial Structure

**Figure 3:**
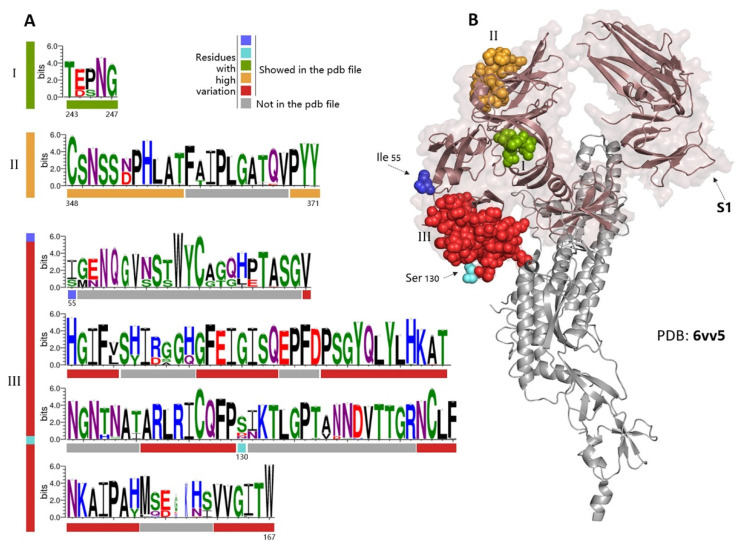
Schematic diagram of the highly mutated regions in PEDV S protein. (**A**) Highly mutated regions in the S protein, analyzed by a sequence logo generator WebLogo 3, were marked with different colors (I, II, and III), and the gray labeled sites indicted the uncontained sites in the PDB 6vv5, and (**B**) Corresponding locations of the highly mutated regions (I, II, and III) in the S protein based on the crystal structure file of PDB 6vv5; the brown transparent labeled area indicted the S1 domain.

The mutation sites in the S protein based on spatial structure were analyzed. According to the results of the mutation assay using 647 sequences of S protein (Figure 3A) and illustrated in the spatial structural data of PEDV S protein (PDB: 6vv5), we refrained from modeling residues showed as gray because these are missing from publicly available structures. It was found that most of the highly mutated residues of S protein were located in the S1 domain, the surface of the S protein (Figure 3B), which was consistent with the assay results of positively selected sites (Figure 1A), indicating that the evolutionary pattern of PEDV S protein is highly S1 domain-related. 

### 3.5. The Evolutionary History of PEDV Lineages

**Figure 4:**
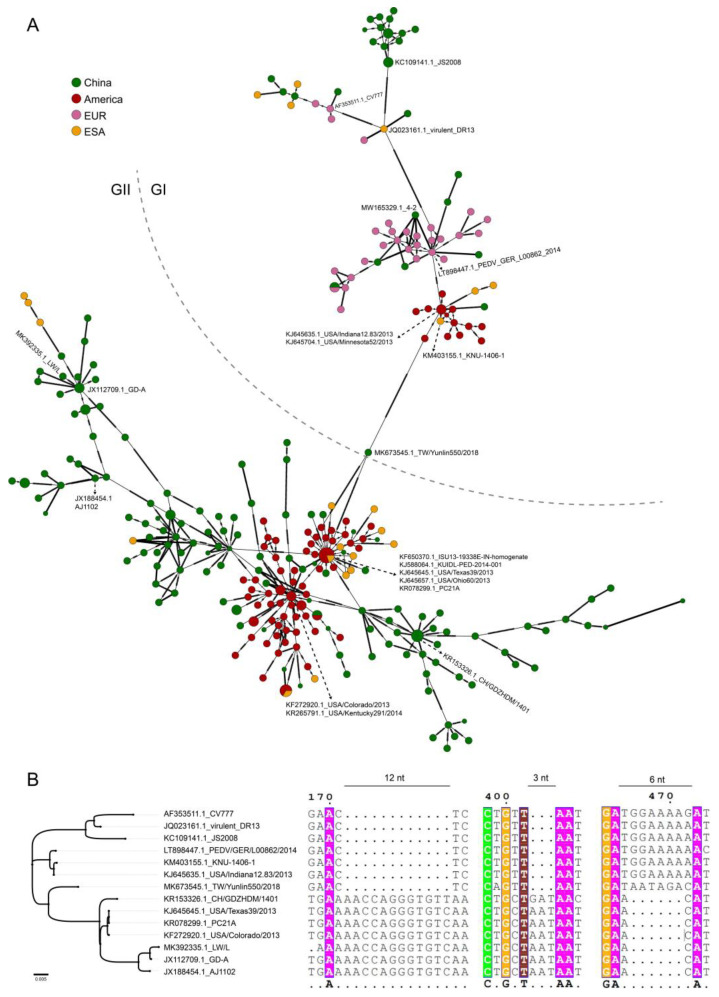
Haplotype analysis of PEDV S gene: (**A**) the haplotype networks of PEDV based on the S gene; different colors represent viruses in different regions; green represents China, red represents the Americas, pink represents Europe (EUR), yellow represents East Asia (except China) and South Asia (ESA); (**B**) evolution of the PEDV based on the representative strains from dominant haplotype; ‘.’ represents the nucleotide sequence with the gap.

In the assay of PEDV lineage formation, the putative lineages of PEDV were found when we constructed the haplotype networks using the PEDV S gene (297 representative strains out of 647 strains). The results showed that the strains were obviously clustered geographically in the lineages (Figure 4A) corresponding to genotype GI and GII, which was consistent with the results of the phylogenetic analysis. We performed alignment analysis on representative strains of the main haplotypes (Figure 4B), and three distinguishable nucleic acid sites among those strains were found, including, a 12 nt insertion of the GII (AACCAGGGTGTC/T), a 3 nt insertion of the GII (G/AAT), and a 6 nt deletion of the GII (GGAAAA, or AATAGA), respectively. Interestingly, the linked strain TW/Yunlin550/2018 (MK673545.1) between GI and GII showed a completely different pattern at the 6 nt site (AATAGA, aa of Asn-Arg), which is in a highly mutated region of the S1 domain as shown in Figure 3 (site III).

## 4. Discussion

The S protein of coronavirus directly interacts with cellular receptors; the diversity of the S gene is often used as the phylogenetic marker in evolutionary analysis. The complete genomes of diverse strains from the global database promotes a better understanding of evolutionary and phylogenetic relationships [31]. In the study of virus evolution, one method of testing for selection is to compute the ratio of nonsynonymous to synonymous substitution rates (ω); ω is expected to have a value of 1 under the assumption of neutral evolution. Positive and negative selection are indicated when ω > 1 and ω < 1, respectively [21]. The M8–M7 comparison model offers a very stringent test of positive selection [32]. In terms of the S gene of different coronaviruses, the M8 model was a better fit than the M7 model; the favor of positive selection here and the high mutation rate of the S gene [14] make it the best target for evolutionary assays, which also was confirmed in the evolutionary comparison of the whole genome sequence with the S gene and other structural protein genes. 

Previous studies showed that natural selection was the main force influencing the codon usage pattern of PEDV, while mutation pressure played a minor role [33,34,35]. Here, if positive selection is the driving force for the higher synonymous substation rate seen in spike, we would expect the FOP of spike to be different from that of the genome. The elevated synonymous substitution rate measured in the S gene might be more likely caused by higher mutation rates, but the FOP of the S gene showed no obvious difference to that of the genomic average; the underlying molecular mechanism remains unclear, and deserves further study. Synonymous substitutions may serve as another layer of genetic regulation, guiding the efficiency of mRNA translation by changing codon usage.

By the application of NEB analysis, we found that S and N proteins were more favored for mutation than other proteins in this study, which was consistent with antigenic study of possible antigenic differences that emerged in both the spike and nucleocapsid proteins between different genogroups [36]. As we showed here, the S gene was found to provide the maximal interpretative power in PEDV evolution because of its high phylogenetic signal with substitution rate, and a phylogenetic topology similar to that obtained from the complete genome [37,38], which would facilitate data analysis.

Variations in the S protein are important for revealing the genetic evolution and the pathogenicity of PEDV strains [39,40,41]. Structurally, we found that the highly mutated residues of the S protein were S1 domain-dominated, indicating a strong S1-related evolutionary pattern of PEDV, which might be induced by the antigenic drift as amino acid positions with significant variation among isolates from different regions and subgroups were found [14]. In other coronaviruses, the naturally occurring spike mutations in NTD influence the infectivity and immunogenicity, and NTD has been identified as one of the mutation hotspots of SARS-CoV-2 [42,43]. PEDV S protein may undergo a conformational change after receptor binding and cleavage by exogenous trypsin, which induces membrane fusion [44].

Selective pressures drive adaptive changes in the coronavirus S proteins directing virus-cell entry. The high hypervariability in the SARS-CoV-2 S protein appears to be driven by counterbalancing pressures of effective virus-cell entry and durable extracellular virus infectivity [45], which could be caused by the variation in amino acid positions. The binding domain of the PEDV cellular receptor APN was shown to reside within a domain in the C-terminal of the S1 domain (residues 477–629), which is close to one of the sites we found in haplotype networks for the potential differentiation of the PEDV genotype. The strain of TW/Yunlin550/2018 (MK673545.1) that is located on the edge of the genotypes of GI and GII showed a completely different pattern at the 6 nt site (AATAGA, aa of Asn-Arg, NR) compared with the GI strains (GGAAAA, aa of Gly-Lys, GK), which might result in better host adaption during virus evolution when facing the counterbalancing pressures; further study on the evolution pattern is needed. 

Geographically, the GI strains in Europe were evolutionarily separated from the GII strains in other global regions. In the genotype GII, the American strains were clustered with most of the strains from Japan and South Korea, and strains in China were shown in a more compact branch. The increasingly international pig industry involves the trade of various breeding materials and animals, which may increase the risk of disease transmission. The global exchange of ingredients has created demand for products that prevent disease transmission from the feed, such as the use of the monoglyceride blend, which could mitigate and prevent PED transmission to piglets from contaminated feed [46].

Overall, we found that S protein showed strong signals of positive selection, and the evolutionary pattern of S protein was highly S1 domain-related, which also represents the marker for clustering lineages corresponding to genotypes GI and GII geographically. These findings provide several fundamental insights into the evolution of PEDV and guidance for developing effective prevention countermeasures against PEDV.

## 5. Conclusions

PEDV S proteins showed strong signals of positive selection, and seven of them were located in the surface of the S protein (S1 domain), suggesting the high selection pressure of S protein. The S gene is more representative at the genome level and the evolutionary pattern is highly S1 domain-related. The three distinguishable nucleic acid sites in the haplotype assay suggested a putative evolutionary mechanism of PEDV. These findings provide several new fundamental insights into the evolution of PEDV and guidance for developing effective prevention countermeasures against PEDV.

## Figures and Tables

**Table 1:** The molecular divergence between PEDV and related viruses.

Gene	Aligned Length (nt)	JS2008	AH2012	AJ1102	OH851	GDS01	PDCoV-NH	SpDCoV_HKU17	TGEV_Purdue_P115	SADS-CoV/GDGL01/2016
Genome	28,033	0.1257 (0.0075 0.0596)	0.0896 (0.0083 0.0926)	0.0889 (0.0085 0.0952)	0.0782 (0.0075 0.0965)	0.0932 (0.0090 0.0961)	0.3278 (0.7386 2.2532)	0.2554 (0.7279 2.8500)	0.1850 (0.4134 2.2343)	0.2039 (0.3713 1.8208)
ORF1a	12,356	0.1493 (0.0082 0.0548)	0.0999 (0.0083 0.0833)	0.1081 (0.0091 0.0842)	0.0949 (0.0084 0.0890)	0.1158 (0.0100 0.0860)	0.5766 (1.0249 1.7774)	0.5470 (1.0125 1.8510)	0.3299 (0.6076 1.8419)	0.2999 (0.4547 1.5160)
ORF1ab	20,346	0.1011 (0.0060 0.0597)	0.0727 (0.0063 0.0872	0.0742 (0.0066 0.0895	0.0668 (0.0061 0.0915)	0.0790 (0.0071 0.0902)	0.3579 (0.7918 2.2122)	0.3232 (0.7881 2.4389)	0.1909 (0.4191 2.1955)	0.1770 (0.3271 1.8478)
S (spike)	4152	0.2352 (0.0183 0.0777)	0.1646 (0.0207 0.1259)	0.1566 (0.0201 0.1282)	0.1378 (0.0180 0.1310)	0.1553 (0.0214 0.1378)	0.2411 (0.5682 2.3568)	/	0.1684 (0.4468 2.6537)	0.5395 (0.8751 1.6220)
ORF3	735	0.5474 (0.0577 0.1055)	0.2019 (0.0196 0.0970)	0.2528 (0.0245 0.0971)	0.2019 (0.0196 0.0970)	0.2528 (0.0245 0.0971)	NA	NA	/	0.4643 (0.7320 1.5766)
M	813	0.2456 (0.0064 0.0261)	0.0715 (0.0043 0.0597)	0.0714 (0.0043 0.0598)	0.0641 (0.0043 0.0666)	0.0714 (0.0043 0.0598)	/	/	0.1688 (0.4790 2.8373)	0.1437 (0.3316 2.3073)
E	303	1.0790 (1.3737 1.2731)	0.0512 (0.0069 0.1342)	0.0786 (0.0069 0.0873)	0.0508 (0.0069 0.1351)	0.1563 (0.0137 0.0880)	/	/	/	/
N	1425	0.0930 (0.0071 0.0762)	0.0918 (0.0142 0.1551)	0.1271 (0.0142 0.1120)	0.0791 (0.0114 0.1439)	0.1143 (0.0128 0.1120)	0.4675 (1.0071 2.1540)	0.4776 (0.9513 1.9918)	/	/

For each gene, the dN/dS (ω) ratio between the PEDV CV777 strain and the other virus is given, and the dN and dS values are given in parenthesis (dN, dS). “/” means that the method of Nei and Gojobori [21] is inapplicable.

## Data Availability

Not applicable.

## Supplementary Materials

The following supporting information can be downloaded at https://www.mdpi.com/article/10.3390/ani12233388/s1. Figure S1: Location and number of the isolated PEDV strains (with qualified sequence data). Figure S2: Positive selection assay of S protein by NEB analysis. Positive selection assay of S protein using the NEB method, the positive selected sites are labeled in red. Figure S3: Amino acid alignment of PEDV S and N proteins. The alignment of the complete amino acid of PEDV S and N proteins was carried out using ESPript 3.0. Table S1: Detailed information of the PEDV complete genomes used in this study. Table S2: Codon usage bias of the 59 sense codons of PEDV CV777.

## References

[1] Pensaert M.B., de Bouck P. (1978). A new coronavirus-like particle associated with diarrhea in swine. Arch. Virol..

[2] Kusanagi K., Kuwahara H., Katoh T., Nunoya T., Ishikawa Y., Samejima T., Tajima M. (1992). Isolation and serial propagation of porcine epidemic diarrhea virus in cell cultures and partial characterization of the isolate. J. Vet. Med. Sci..

[3] Choi J.C., Lee K.K., Pi J.H., Park S.Y., Song C.S., Choi I.S., Lee J.B., Lee D.H., Lee S.W. (2014). Comparative genome analysis and molecular epidemiology of the reemerging porcine epidemic diarrhea virus strains isolated in Korea. Infect. Genet. Evol..

[4] Li W., Li H., Liu Y., Pan Y., Deng F., Song Y., Tang X., He Q. (2012). New variants of porcine epidemic diarrhea virus, China, 2011. Emerg. Infect. Dis..

[5] Yang X., Huo J.Y., Chen L., Zheng F.M., Chang H.T., Zhao J., Wang X.W., Wang C.Q. (2013). Genetic variation analysis of reemerging porcine epidemic diarrhea virus prevailing in central China from 2010 to 2011. Virus Genes.

[6] Chen J., Wang C., Shi H., Qiu H., Liu S., Chen X., Zhang Z., Feng L. (2010). Molecular epidemiology of porcine epidemic diarrhea virus in China. Arch. Virol..

[7] Stevenson G.W., Hoang H., Schwartz K.J., Burrough E.R., Sun D., Madson D., Cooper V.L., Pillatzki A., Gauger P., Schmitt B.J. (2013). Emergence of porcine epidemic diarrhea virus in the United States: Clinical signs, lesions, and viral genomic sequences. J. Vet. Diagn. Invest..

[8] Vlasova A.N., Marthaler D., Wang Q., Culhane M.R., Rossow K.D., Rovira A., Collins J., Saif L.J. (2014). Distinct characteristics and complex evolution of PEDV strains, North America, May 2013–February 2014. Emerg. Infect. Dis..

[9] Chang S.H., Bae J.L., Kang T.J., Kim J., Chung G.H., Lim C.W., Laude H., Yang M.S., Jang Y.S. (2002). Identification of the epitope region capable of inducing neutralizing antibodies against the porcine epidemic diarrhea virus. Mol. Cells.

[10] Wicht O., Li W., Willems L., Meuleman T.J., Wubbolts R.W., van Kuppeveld F.J., Rottier P.J., Bosch B.J. (2014). Proteolytic activation of the porcine epidemic diarrhea coronavirus spike fusion protein by trypsin in cell culture. J. Virol..

[11] Kang K.J., Kim D.H., Hong E.J., Shin H.J. (2021). The Carboxy Terminal Region on Spike Protein of Porcine Epidemic Diarrhea Virus (PEDV) Is Important for Evaluating Neutralizing Activity. Pathogens.

[12] Chang C.Y., Wang Y.S., Wu J.F., Yang T.J., Chang Y.C., Chae C., Chang H.W., Hsu S.D. (2021). Generation and Characterization of a Spike Glycoprotein Domain A-Specific Neutralizing Single-Chain Variable Fragment against Porcine Epidemic Diarrhea Virus. Vaccines.

[13] Wang L., Byrum B., Zhang Y. (2014). New variant of porcine epidemic diarrhea virus, United States, 2014. Emerg. Infect. Dis..

[14] Tang X., Wu C., Li X., Song Y., Yao X., Wu X., Duan Y., Zhang H., Wang Y., Qian Z. (2020). On the origin and continuing evolution of SARS-CoV-2. Natl. Sci. Rev..

[15] Guo J., Fang L., Ye X., Chen J., Xu S., Zhu X., Miao Y., Wang D., Xiao S. (2019). Evolutionary and genotypic analyses of global porcine epidemic diarrhea virus strains. Transbound. Emerg. Dis..

[16] Lee D.K., Park C.K., Kim S.H., Lee C. (2010). Heterogeneity in spike protein genes of porcine epidemic diarrhea viruses isolated in Korea. Virus Res..

[17] Edgar R.C. (2004). MUSCLE: Multiple sequence alignment with high accuracy and high throughput. Nucleic Acids Res..

[18] Capella-Gutierrez S., Silla-Martinez J.M., Gabaldon T. (2009). trimAl: A tool for automated alignment trimming in large-scale phylogenetic analyses. Bioinformatics.

[19] Stamatakis A. (2014). RAxML version 8: A tool for phylogenetic analysis and post-analysis of large phylogenies. Bioinformatics.

[20] Darriba D., Posada D., Kozlov A.M., Stamatakis A., Morel B., Flouri T. (2020). ModelTest-NG: A New and Scalable Tool for the Selection of DNA and Protein Evolutionary Models. Mol. Biol. Evol..

[21] Gao F., Chen C., Arab D.A., Du Z., He Y., Ho S.Y.W. (2019). EasyCodeML: A visual tool for analysis of selection using CodeML. Ecol. Evol..

[22] Nei M., Gojobori T. (1986). Simple methods for estimating the numbers of synonymous and nonsynonymous nucleotide substitutions. Mol. Biol. Evol..

[23] Yang Z., Wong W.S., Nielsen R. (2005). Bayes empirical bayes inference of amino acid sites under positive selection. Mol. Biol. Evol..

[24] Scheffler K., Seoighe C. (2005). A Bayesian model comparison approach to inferring positive selection. Mol. Biol. Evol..

[25] Robert X., Gouet P. (2014). Deciphering key features in protein structures with the new ENDscript server. Nucleic Acids Res..

[26] Crooks G.E., Hon G., Chandonia J.M., Brenner S.E. (2004). WebLogo: A sequence logo generator. Genome Res..

[27] Kirchdoerfer R.N., Bhandari M., Martini O., Sewall L.M., Bangaru S., Yoon K.J., Ward A.B. (2021). Structure and immune recognition of the porcine epidemic diarrhea virus spike protein. Structure.

[28] Rozas J., Ferrer-Mata A., Sanchez-DelBarrio J.C., Guirao-Rico S., Librado P., Ramos-Onsins S.E., Sanchez-Gracia A. (2017). DnaSP 6: DNA Sequence Polymorphism Analysis of Large Data Sets. Mol. Biol. Evol..

[29] Leigh J.W., Bryant D. (2015). popart: Full-feature software for haplotype network construction. Genet. Mol. Res..

[30] Kumar S., Stecher G., Li M., Knyaz C., Tamura K. (2018). MEGA X: Molecular Evolutionary Genetics Analysis across Computing Platforms. Mol. Biol. Evol..

[31] Jarvis M.C., Lam H.C., Zhang Y., Wang L., Hesse R.A., Hause B.M., Vlasova A., Wang Q., Zhang J., Nelson M.I. (2016). Genomic and evolutionary inferences between American and global strains of porcine epidemic diarrhea virus. Prev. Vet. Med..

[32] Anisimova M., Bielawski J.P., Yang Z. (2001). Accuracy and power of the likelihood ratio test in detecting adaptive molecular evolution. Mol. Biol. Evol..

[33] Yu X., Liu J., Li H., Liu B., Zhao B., Ning Z. (2021). Comprehensive analysis of synonymous codon usage patterns and influencing factors of porcine epidemic diarrhea virus. Arch. Virol..

[34] Xu X., Li P., Zhang Y., Wang X., Xu J., Wu X., Shen Y., Guo D., Li Y., Yao L. (2019). Comprehensive analysis of synonymous codon usage patterns in orf3 gene of porcine epidemic diarrhea virus in China. Res. Vet. Sci..

[35] Chen Y., Shi Y., Deng H., Gu T., Xu J., Ou J., Jiang Z., Jiao Y., Zou T., Wang C. (2014). Characterization of the porcine epidemic diarrhea virus codon usage bias. Infect. Genet. Evol..

[36] Kim S.J., Nguyen V.G., Huynh T.M., Park Y.H., Park B.K., Chung H.C. (2020). Molecular Characterization of Porcine Epidemic Diarrhea Virus and Its New Genetic Classification Based on the Nucleocapsid Gene. Viruses.

[37] Sung M.H., Deng M.C., Chung Y.H., Huang Y.L., Chang C.Y., Lan Y.C., Chou H.L., Chao D.Y. (2015). Evolutionary characterization of the emerging porcine epidemic diarrhea virus worldwide and 2014 epidemic in Taiwan. Infect. Genet. Evol..

[38] Sun M., Ma J., Wang Y., Wang M., Song W., Zhang W., Lu C., Yao H. (2015). Genomic and epidemiological characteristics provide new insights into the phylogeographical and spatiotemporal spread of porcine epidemic diarrhea virus in Asia. J. Clin. Microbiol..

[39] Wang X., Chen J., Shi D., Shi H., Zhang X., Yuan J., Jiang S., Feng L. (2016). Immunogenicity and antigenic relationships among spike proteins of porcine epidemic diarrhea virus subtypes G1 and G2. Arch. Virol..

[40] Fan B., Jiao D., Zhao X., Pang F., Xiao Q., Yu Z., Mao A., Guo R., Yuan W., Zhao P. (2017). Characterization of Chinese Porcine Epidemic Diarrhea Virus with Novel Insertions and Deletions in Genome. Sci. Rep..

[41] Sun J., Li Q., Shao C., Ma Y., He H., Jiang S., Zhou Y., Wu Y., Ba S., Shi L. (2018). Isolation and characterization of Chinese porcine epidemic diarrhea virus with novel mutations and deletions in the S gene. Vet. Microbiol..

[42] Peng Q., Zhou R., Liu N., Wang H., Xu H., Zhao M., Yang D., Au K.K., Huang H., Liu L. (2022). Naturally occurring spike mutations influence the infectivity and immunogenicity of SARS-CoV-2. Cell. Mol. Immunol..

[43] Jaru-Ampornpan P., Jengarn J., Wanitchang A., Jongkaewwattana A. (2017). Porcine Epidemic Diarrhea Virus 3C-Like Protease-Mediated Nucleocapsid Processing: Possible Link to Viral Cell Culture Adaptability. J. Virol..

[44] Park J.E., Cruz D.J., Shin H.J. (2011). Receptor-bound porcine epidemic diarrhea virus spike protein cleaved by trypsin induces membrane fusion. Arch. Virol..

[45] Qing E., Kicmal T., Kumar B., Hawkins G.M., Timm E., Perlman S., Gallagher T. (2021). Dynamics of SARS-CoV-2 Spike Proteins in Cell Entry: Control Elements in the Amino-Terminal Domains. mBio.

[46] Phillips F.C., Rubach J.K., Poss M.J., Anam S., Goyal S.M., Dee S.A. (2021). Monoglyceride reduces viability of porcine epidemic diarrhea virus in feed and prevents disease transmission to post-weaned piglets. Transbound. Emerg. Dis..

