# Supplementary material for: Molecular and Structural Evolution of Porcine Epidemic Diarrhea Virus
Source: Animals (Basel). 2022 Dec 1;12(23):3388. doi: 10.3390/ani12233388 (PMC9736354; doi:10.3390/ani12233388)
Supplement: Supplementary file 1 [file animals-12-03388-s001.zip › Supplementary Figure S3 S-N aa alin.pdf]

```
                         1         10        20        30              40        50      60            70
CV777-AF353511-GI-a      MRSLIYFWLLLPVLPTLSLPQDVTRCQSTTNFRRFFSKF......NVQAPAVVVLGGYLPSM....NSSSWYCGTGIETA
JS2008-KC210146-GI-b     MTPLIYFWLFLPVLLTLSLPQDVTRCQSTINFRRFFSKF......NVQAPAVVVLGGYLPSM....NSSSWYCGTGIETD
AH2012-KC210145-GII-a    MKSLTYFWLFLPVLSTLSLPQDVTRCSANTNFRRFFSKF......NVQAPAVVVLGGYLPIGENQGVNSTWYCAGQHPTA
AJ1102-JX188454-GII-b    MKSLTYFWLFLPVLSTLSLPQDVTRCSANTNFRRFFSKF......NVQAPAVVVLGGYLPIGENQGVNSTWYCAGQHPTA
OH851-KJ399978-GII-c     MKSLNYFWLFLPVLSTLSLPQDVTRCQSTINFRRFFSKF......NVQAPAVVVLGGYLPSM....NSSSWYCGTGLETA
GDS01-KM089829-GII-b     MKSLTYFWLFLPVLSTLSLPQDVTRCSANTNFRRFFSKF......NVQAPAVVVLGGYLPIGENQGVNSTWYCAGQHPTA
PDCoV-NH-KU981059        MQRALLIMTLLCL...................................................................
SpDCoV_HKU17             MQGALLITFIAVV...................................................................
TGEV_Purdue_P115         MKKLFVVLVVMPLIYGDNFPCSKLTNRTIGNQWNLIETFLLNYSSRLPPNSDVVLGDYFPTVQPWFNCIRNDSNDLYVTL
SADS-CoV/GDGL01/2016     MKLFTVFTLLASI......................................RVLYG........................
                         1   5    10   15   20   25   30   35   40   45   50   55   60   65   70   75   80

                                   80        90        100       110       120       130       140
CV777-AF353511-GI-a      SGVHGIFLSYIDSGQGFEIGISQE.PFDPSGYQLYLHKATNGNTNAIARLRICQFPDNKTLGPTV.NDVTTGRNCLFNKA
JS2008-KC210146-GI-b     SGVHGIFLSYIDSGQGFEIGISQE.PFDPSGYQLYLHKATNGNTSAIARLRICQFPDNKTLGPTV.NDVTTGRNCLFNKA
AH2012-KC210145-GII-a    SGVHGIFVSHIRGGHGFEIGISQE.PFDPSGYQLYLHKATNGNTNATARLRICQFPSIKTLGPTANNDVTTGRNCLFNKA
AJ1102-JX188454-GII-b    SGVHGIFLSHIRGGHGFEIGISQE.PFDPSGYQLYLHKATNGNTNATARLRICQFPSIKTLGPTANNDVTTGRNCLFNKA
OH851-KJ399978-GII-c     SGVHGIFLSYIDAGQGFEIGISQE.PFDPSGYQLYLHKATNGNHNAIARLRICQFPDNKTLGPTV.NDVTTGRNCLFNKA
GDS01-KM089829-GII-b     SGVHGIFLSHIRGGHGFEIGISQE.PFDPSGYQLYLHKATNGNTNATARLRICQFPSIKTLGPTANNDVTTGRNCLFNKA
PDCoV-NH-KU981059        ....................................................VRAK........................
SpDCoV_HKU17             ....................................................TSSP........................
TGEV_Purdue_P115         ENLKAVYWDYATENITWNHRQRLNVVVNGYPYSITVTTTRNFNSAEGAIICICKGSPPTTTTESS.LTCNWGSECRLNHK
SADS-CoV/GDGL01/2016     ....................................................CESVDFN................LFNTI
                             85   90   95   100  105  110  115  120  125  130  135  140  145  150  155  160

                         150        160       170       180       190       200                 210
CV777-AF353511-GI-a      IPAYMRDGK....DIVVGITWDNDRVTVFADKI.YHFYLKNDWSRVATRCYNRRSCA...........MQYVYTPTYYML
JS2008-KC210146-GI-b     IPA.LQDGK....NIVVGITWDNDRVTVFADKI.YHFYIKNDWSRVATRCYNKRSCA...........MQYVYTPTYYML
AH2012-KC210145-GII-a    IPAHMSEHS......VVGITWDNDRVTVFSDKI.YYFYFKNDWSRVATKCYNSGGCA...........MQYVYEPTYYML
AJ1102-JX188454-GII-b    IPAHMSEHS......VVGITWDNDRVTVFSDKI.YYFYFKNDWSRVATKCYNSGGCA...........MQYVYEPTYYML
OH851-KJ399978-GII-c     IPAYMQDGK....NIVVGITWDNDRVTVFADKI.YHFYLKNDWSRVATRCYNKRSCA...........MQYVYTPTYYML
GDS01-KM089829-GII-b     IPAHMSEHS......VVGITWDNDRVTVFSDKI.YYFYFKNDWSRVATKCYNSGGCA...........MQYVYEPTYYIL
PDCoV-NH-KU981059        ............................FADDL.LDLL...........TFPGA..............HRFLHKLTSNSS
SpDCoV_HKU17             ............................LADKI.LDFL...........TFPEA..............HAYLFPQSRMVR
TGEV_Purdue_P115         FPICPSNSEANCGNMLYGLQWFADEVVAYLHGASYRISFENQWSGTVTFGDMRATTLEVAGTLVDLWWFNPVYDVSYYRV
SADS-CoV/GDGL01/2016     FSTH................RGLSNTTSVITGA.YPSTNKSDWS.....CNTRT..........................
                             165  170  175  180  185  190  195  200  205  210  215  220  225  230  235  240

                                   220       230       240       250       260       270       280
CV777-AF353511-GI-a      NVTSAGEDGIYYEPCTANCTGYAANVFATDSNGHIPEGFSFNNWFLLSNDSTLLHGKVVSNQPLLVN...CLLAIPKIYG
JS2008-KC210146-GI-b     NVTSAGEDGIYYEPCTANCSGYAANVFATDSNGHIPEGFSFNNWFLLSNDSTLLHGKVVSNQPLLVN...CLWAIPKIYG
AH2012-KC210145-GII-a    NVTSAGEDGISYQPCTANCIGYAANVFATEPNGHIPEGFSFNNWFLLSNDSTLVHGKVVSNQPLLVN...CLLAIPKIYG
AJ1102-JX188454-GII-b    NVTSAGEDGISYQPCTANCIGYAANVFATEPNGHIPEGFSFNNWFLLSNDSTLVHGKVVSNQPLLVN...CLLAIPKIYG
OH851-KJ399978-GII-c     NVTSAGEDGIYYEPCTANCSGYAANVFATDSNGHIPEGFSFNNWFLLSNDSTLLHGKVVSNQPLLVN...CLLAIPKIYG
GDS01-KM089829-GII-b     NVTSAGEDGISYQPCTANCIGYAANVFATEPNGHIPEGFSFNNWFLLSNDSTLVHGKVVSNQPLLVN...CLLAIPKIYG
PDCoV-NH-KU981059        S.............HYSRANNFDVGVL....PGYPTKNVNL..FSPLTNSTLPINGLHRSYQPLMLN...CLTKITNHTL
SpDCoV_HKU17             NIEGG.........ATERC......MYVQE.GGFIPDNFTFPQWFVLTNDSTLLQGELTTSQPLIVNGHLCSWQSNQQY.
TGEV_Purdue_P115         NNKNG...TTVVSNCTDQCASYVANVFTTQPGGFIPSDFSFNNWFLLTNSSTLVSGKLVTKQPLLVN...CLWPVPSFEE
SADS-CoV/GDGL01/2016     ..............GHLSGSGFGIGLYVQTPR....EQYQY........DGSGAGGYTIAVSPIHVTN..LTWEL.....
                             245  250  255  260  265  270  275  280  285  290  295  300  305  310  315  320

                        290         300        310       320       330       340
CV777-AF353511-GI-a      LGQFFSFNHT..........MDGVCNGAAV.......DRAPEALRFNIN.DTSVILAEG..SIVLHTALGTNLSFVCS.N
JS2008-KC210146-GI-b     LGQFFSFNQT..........MDGVCNGAAV.......QRAPEALRFNIN.DTFVILAEG..SIVLHTALGTNLSFVCS.N
AH2012-KC210145-GII-a    LGQFFSFNQT..........IDGVCNGAAV.......QRAPEALRFNIN.DTSVILAEG..SIVLHTALGTNFSFVCS.N
AJ1102-JX188454-GII-b    LGQFFSFNQT..........IDGVCNGAAV.......QRAPEALRFNIN.DTSVILAEG..SIVLHTALGTNFSFVCS.N
OH851-KJ399978-GII-c     LGQFFSFNQT..........MDGVCNGAAA.......QRAPEALRFNIN.DTSVILAEG..SIVLHTALGTNLSFVCS.N
GDS01-KM089829-GII-b     LGQFFSFNQT..........IDGVCNGAAV.......QRAPEALRFNIN.DTSVILAEG..SIVLHTALGTNFSFVCS.N
PDCoV-NH-KU981059        SMYLLPSEIQ..........TYS.CGGAMV......KHQTHDAVRIILDLTATDHISVE..VVGQH...GENYVFVCSEQ
SpDCoV_HKU17             ..RKYSFNTSCPLPADGCNPTWG.SGNLKLFKGWNNTKGLTDNIRINVN.ISQSDYRTSAGSVGLELEGGGTVNITCT.N
TGEV_Purdue_P115         AASTFCFEGA..........GFDQCNGAVL.......NNTVDVIRFNLNFTTNVQSGKGATVFSLNTTGGVTLEISCYTV
SADS-CoV/GDGL01/2016     ......WIHR..........KWGVNSVVTV........RLCRWWQFMSF.NSTSHAADA..........GPTNAFECLIN
                             325  330  335  340  345  350  355  360  365  370  375  380  385  390  395  400

                         350       360       370       380       390       400       410       420
CV777-AF353511-GI-a      SSDPHLAIFAIPLGATEV.PYYCFLKVDTYNSTVYKFLAVLPPTVREIVITKYGDVYVNGFGYLHLGLLDAVTINFTGHG
JS2008-KC210146-GI-b     SSDPHKAIFTIPLGVTEV.PYYCFLKVDTYKSTVYKFLAVLPPTVKEIVITKYGDVYVNGFGYLHLGLLDAVTINFTGHG
AH2012-KC210145-GII-a    SSNPHLATFAIPLGATQV.PYYCFLKVDTYNSTVYKFLAVLPPTVREIVITKYGDVYVNGFGYLHLGLLDAVTINFTGHG
AJ1102-JX188454-GII-b    SSDPHLATFAIPLGAIQV.PYYCFLKVDTYNSTVYKFLAVLPPTVREIVITKYGDVYVNGFGYLHLGLLDAVTVNFTGHG
OH851-KJ399978-GII-c     SSDPHLATFAIPLGATQV.PYYCFLKVDTYNSTVYKFLAVLPPTVREIVITKYGDVYVNGFGYLHLGLLDAVTINFTGHG
GDS01-KM089829-GII-b     SSEPHLATFAIPLGAIQV.PYYCFLKVDTYNSTVYKFLAVLPPTVREIVITKYGDVYVNGFGYLHLGLLDAVTINFTGHG
PDCoV-NH-KU981059        FNYTTALHNSTFFSLNS..ELYCFTN........NTYLGILPPDLTDFTVYRTGQFYANGYLLGTLPITVNYVRLYRGHL
SpDCoV_HKU17             SSTPVTTYSLLPWARSSGEPIYCFANVSSPSQSYIDFMGILPPFVSEIAFDRSGSIYINGYRYFKISPLLNVELQLNFNL
TGEV_Purdue_P115         SDSSFFSYGEIPFGVTDG.PRYCYVH...YNGTALKYLGTLPLSVKEIAISKWGHFYINGYNFFSTFPIDCISFNLTTGD
SADS-CoV/GDGL01/2016     GSYPTHRNTGYMFGV....TWY.........NDLVRI..VFPPTVLEMQL........DGLQWERVQFNSPVN...AGHA
                             405  410  415  420  425  430  435  440  445  450  455  460  465  470  475  480
```

```
                      430       440       450       460       470       480       490       500
CV777-AF353511-GI-a   TDDDVSGFWTIASTNFVDALIEVQ.GTSIQRILYCDDP.VSQLKCSQVAFDLDDGFYPISSRNLLSHEQPISFVTL....
JS2008-KC210146-GI-b  TDDDVSGFWTVASTNFVDALIEVQ.GTAIQRILYCDDP.VSQLKCSQVSFDLDDGFYPISSRNLLSHEQPISFVTL....
AH2012-KC210145-GII-a TDDDVSGFWTIASTNFVDALIEVQ.GTAIQRILYCDDP.VSQLKCSQVAFDLDDGFYPISSRNLLSHEQPISFVTL....
AJ1102-JX188454-GII-b TDDDVSGFWTIASTNFVDALIEVQ.GTAIQRILYCDDP.VSQLKCSQVSFDLDDGFYPISSTNLLSHEQPTSFVTL....
OH851-KJ399978-GII-c  TDDDVSGFWTIASTNFVDALIEVQ.GTAIQRILYCDDP.VSQLKCSQVAFDLDDGFYPISSRNLLSHEQPISFVTL....
GDS01-KM089829-GII-b  TDDDVSGFWTIASTNFVDALIEVQ.GTAIQRILYCDDP.VSQLKCSQVAFDLDDGFYRISSTNLLSHEQPTSFVTL....
PDCoV-NH-KU981059     SANS....AHFALANLTDTLITLT.NTTISQITYCDKSVVDSIACQRSSHEVEDGFYSDPKSAV..RARQRTIVTL....
SpDCoV_HKU17          TSD....HFSVTWSNYTEVHLNTT.NGYIHQIKYCYDP.LDKLACEMNTFQLPDGVYPYTPAQQ..QALPETFVTT....
TGEV_Purdue_P115      SDV....FWTIAYTSYTEALVQVE.NTAITKVTYCNSH.VNNIKCSQITANLNNGFYPVSSSEV..GLVNKSVVLL....
SADS-CoV/GDGL01/2016  TR......FNVV.KDISTVLVETNSGGSVFRYSYCADGFVNGLQCKLRLFDIPPGVYSNSEVEY.....PTALYTVVHNM
                        485  490  495  500  505  510  515  520  525  530  535  540  545  550  555  560

                                510                 520       530       540                 550
CV777-AF353511-GI-a   ...PSFNDHSFV..NITVS.....AAFGGLSSANLVASDTTINGFSS..FCVDTRQFTI........TLFYNV.....TN
JS2008-KC210146-GI-b  ...PSFNDHSFV..NITVS.....AAFGGHSGANLIASDTTINGFSS..FCVDTRQFTI........TLFYNV.....TN
AH2012-KC210145-GII-a ...PSFNDHSFV..NITVS.....ASFGGHSGANLIASDTTINGFSS..FCVDTRQFTI........SLFYNV.....TN
AJ1102-JX188454-GII-b ...PSFNDHSFV..NITVS.....AAFGGHSGANLIASDTTINGFSS..FCVDTRQFTI........SLFYNV.....TN
OH851-KJ399978-GII-c  ...PSFNDHSFV..NITVS.....ASFGGHSGANLIASDTTINGFSS..FCVDTRQFTI........SLFYNV.....TN
GDS01-KM089829-GII-b  ...PSFNDHSFV..NITVS.....AAFGGHSGANLIASDTTINGFSS..FCVDTRQFTI........SLFYNV.....TN
PDCoV-NH-KU981059     ...PKLPELEVVQLNISAH.....MDFGEARLDSVT.....INGNTS..YCVTKPYFRL........ETNFMC.......
SpDCoV_HKU17          ...PVYANHTTV..VVRTQYTVSSTGINGPPSWSTVELDGAIND..T..LCVNSRQFTVHLNT....TVHYTA.....AH
TGEV_Purdue_P115      ...PSFYTHTIV..NITIGLGMKRSGYGQPIASTLSNITLPMQDHNTDVYCIRSDQFSVYVHSTCKSALWDNIFKRNCTD
SADS-CoV/GDGL01/2016  SACPERPD....SYCGSNS.....CPFKRAVFSNCI.....VNYTTW..VNPDQRDFQH........LILPNGKFNPFTE
                        565  570  575  580  585  590  595  600  605  610  615  620  625  630  635  640

                      560       570       580       590       600       610       620       630
CV777-AF353511-GI-a   SYGYVSKSQDSNCPFTLQSVNDYLSFSKFCVSTSLLAGACTIDLFGYPAFGSGVKLTSLYFQFTKGELITGTPKPLEGIT
JS2008-KC210146-GI-b  SYGYVSKSQDSNCPFTLQSVNDYLSFSKFCVSTSLLAGACTIDLFGYPEFGSGVKFTSLYFQFTKGELITGTPKPLQGVT
AH2012-KC210145-GII-a SYGYVSNSQDSNCPFTLQSVNDYLSFSKFCVSTSLLASACTIDLFGYPDFGSGVKFTSLYFQFTKGELITGTPKPLEGVT
AJ1102-JX188454-GII-b SYGYVSKSQDSNCPFTLQSVNDYLSFSKFCVSTSLLASACTIDLFGYPEFGSGVKFTSLYFQFTKGELITGTPKPLEGVT
OH851-KJ399978-GII-c  SYGYVSKSQDSNCPFTLQSVNDYLSFSKFCVSTSLLASACTIDLFGYPEFGSGVKFTSLYFQFTKGELITGTPKPLEGVT
GDS01-KM089829-GII-b  SYGYVSKSQDSNCPFTLQSVNDYLSFSKFCVSTSLLASACTIDLFGYPEFGSGVKFTSLYFQFTKGELITGTPKPLEGVT
PDCoV-NH-KU981059     .TGCTMNLRTDTCSFDLSAVNNGMSFSQFCLST..ESGACEMKIIVTYVWNYLLRQR.LYVTAVEGQTHTGT..TSVHAT
SpDCoV_HKU17          LFG..TEFVAGTCPFTLPNINNYLTFGSICFST.VNNGGCTIHVQKVLRH.YRYTFGTIYVSYQPGNQITAMPKASSGTT
TGEV_Purdue_P115      VLDATAVIKTGTCPFSFDKLNNYLTFNKFCLSLSPVGANCKFDVAARTRTNEQV.VRSLYVIYEEGDNIVGVPSDNSGVH
SADS-CoV/GDGL01/2016  CNG.LNRIVDGCVPGFVLRVGRGKAVNRTIVTPYLKPYEC........FGWSWNDNQDSIYDWWIADFVSTG...AFVCES
                        645  650  655  660  665  670  675  680  685  690  695  700  705  710  715  720

                      640       650       660       670       680       690       700       710
CV777-AF353511-GI-a   DVSFMTLDVCTKYTIYGFKGEGIITLTNSSILAGVYYTSD.SGQLLAFKNVTSGAVYSVTPC.SFSEQAAYVNDDIVGVI
JS2008-KC210146-GI-b  DVSFMTLDVCTKYTIYGFKGEGIITLTNSSFLAGVYYTSD.SGQLLAFKNVTSGAVYSVTPC.SFSEQAAYVDDDIVGVI
AH2012-KC210145-GII-a DVSFMTLDVCTKYTIYGFKGEGIITLTNSSFLAGVYYTSD.SGQLLAFKNVTSGAVYSVTPC.SFSEQAAYVDDDIVGVI
AJ1102-JX188454-GII-b DVSFMTLDVCTKYTIYGFKGEGIITLTNSSFLAGFYYTSD.SGQLLAFKNVTSGAVYSVTPC.SFSEQAAYVDDDIVGVI
OH851-KJ399978-GII-c  DVSFMTLDVCTKYTIYGFKGEGIITLTNSSFLAGVYYTSD.SGQLLAFKNVTSGDVYSVTPC.SFSEQAAYVDDDIVGVI
GDS01-KM089829-GII-b  DVSFMTLDVCTKYTIYGFKGEGIITLTNSSFLAGVYYTSD.SGQLLAFKNVTSGAVYSVTPC.SFSEQAAYVDDDIVGVI
PDCoV-NH-KU981059     DTSSVITDVCTDYTIYGVSGTGIIKPSDLLLHNGIAFTSP.TGELYAFKNITTGKTLQVLPC.ETPSQLIVINNTVVGAI
SpDCoV_HKU17          DISTVYLNVCTKYNIYGKTGTGVITKTNETHIGGLYYSSL.SGDLLAFKNSTTQTIYSITPC.ELSAQVAVYNDSIIAAF
TGEV_Purdue_P115      DLSVLHLDSCTDYNIYGRTGVGIIRQTNRTLLSGLYYTSL.SGDLLGFKNVSDGVIYSVTPC.DVSAQAAVIDGTIVGAI
SADS-CoV/GDGL01/2016  NPEAPKTGVCVTYTVEKVTFQGVLYESNFTF..AQYYNLLYVGSQLRYVRIL.GKVYEVSSCFEASYDVLYRNNQSFGLL
                        725  730  735  740  745  750  755  760  765  770  775  780  785  790  795  800

                                          720       730                     740       750       760
CV777-AF353511-GI-a   .............SSLSN...STFNNTRELPGFFYHSN..............DGSNCTEPVLVYSNIGVCKS..GSIGY
JS2008-KC210146-GI-b  .............SSLSN...STFNNTRELPGFFYHSN..............DGSNCTEPVLVYSNIGVCKS..GSIGY
AH2012-KC210145-GII-a .............SSLSN...STFNSTRELPGFFYHSN..............DGSNCTEPVLVYSNIGVCKS..GSIGY
AJ1102-JX188454-GII-b .............SSLSS...STFNSTRELPGFFYHSN..............DGSNCTEPVLVYSNIGVCKS..GSIGY
OH851-KJ399978-GII-c  .............SSLSS...STFNSTRELPGFFYHSN..............DGSNCTEPVLVYSNIGVCKS..GSIGY
GDS01-KM089829-GII-b  .............SSLSS...STFNSTRELPGFFYHSN..............DGSNCTEPVLVYSNIGVCKS..GSIGY
PDCoV-NH-KU981059     .............TSSNSTENNRFTTTIVTPTFFYSTNA.............TTFNCTKPVLSYGPISVCSD..GAIAG
SpDCoV_HKU17          .............TSTENFTFSDFSYKLKTPMFYYHSI..............GNTTCEAPAITFGSIGVCPD..GGLII
TGEV_Purdue_P115      .............TSINS.ELLGLTHWTTTPNFYYYSIYNYTNDRTRGTAIDSNDVDCE.PVITYSNIGVCKN..GAFVF
SADS-CoV/GDGL01/2016  YRSFDCNQLYIKSARFVDRLLPSHNGTATVLGCLFNASYAPND.........TMVNCTNPL....GDGFCADLLGNVAV
                        805  810  815  820  825  830  835  840  845  850  855  860  865  870  875  880

                             770       780       790       800       810       820       830
CV777-AF353511-GI-a   VPSQYGQVK...IAPTVTGNIS.IPTNFSMSIRTEYLQLYNTPVSVDCATYVCNGNSRCKQLLTQYTAACKTIESALQLS
JS2008-KC210146-GI-b  VPSQSGQVK...IAPTVTGNIS.IPTNFSMSIRTEYLQLYNTPVSVDCVTYVCNGNSRCKQLLTQYTAACKTIESALQLS
AH2012-KC210145-GII-a VPSQSGQVK...IAPTVTGNIS.IPTNFSMSIRTEYLQLYNTPVSVDCATYVCNGNSRCKQLLTQYTAACKTIESALQLS
AJ1102-JX188454-GII-b VPSQSGQVK...IAPTVTGNIS.IPTNFSMSIRTEYLQLYNTPVSVDCATYVCNGNSRCKQLLTQYTAACKTIESALQLS
OH851-KJ399978-GII-c  VPSQSGQVK...IAPTVTGNIS.IPTNFSMSIRTEYLQLYNTPVSVDCATYVCNGNSRCKQLLTQYTAACKTIESALQLS
GDS01-KM089829-GII-b  VRSQSGQVK...IAPTVTGNIS.IPTNFSMSIRTEYLQLYNTPVIVDCATYVCNGNSRCKQLLTQYTAACKTIESALQLS
PDCoV-NH-KU981059     TSTLQNTRPS..IVSLYDGEVE.IPSAFSLSVQTEYLQVQAEQVIVDCPQYVCNGNSRCLQLLAQYTSACSNIEAALHSS
SpDCoV_HKU17          QSATTNDVDA..VVPISTQNIS.IPINFTVSIQTEYIQIEHHPITVDCRKYVCNGNPRCLQLLLQYTSACSTIEQALALN
TGEV_Purdue_P115      INVTHSDGD...VQPISTGNVT.IPTNFTISVQVEYIQVYTTPVSIDCSRYVCNGNPRCNKLLTQYVSACQTIEQALAMG
SADS-CoV/GDGL01/2016  RRMTFEKHDTTYVAPVTNERYTEMPLDHQLILTEQFLQTTMPKFSVSCETYICDVSKACKNLLFRYGGFCQKVEADIRGA
                        885  890  895  900  905  910  915  920  925  930  935  940  945  950  955  960
```

```
                       840       850       860       870       880       890       900
CV777-AF353511-GI-a    ARLESVEVNSMLTI..SEEALQLATISSFNG....DG..YNFTNVLGASVYD.....PASGRVVQKRSVIEDLLFNKVVT
JS2008-KC210146-GI-b   ARLESVEVNSMLTI..SEEALQLATISSFNG....DG..YNFTNVLGVSVYD.....PASGRVVQKGSFIEDLLFNKVVT
AH2012-KC210145-GII-a  ARLESVEVNSMLTI..SEEALQLATISSFNG....DG..YNFTNVLGVSVYD.....PASGRVVQKRSFIEDLLFNKVVT
AJ1102-JX188454-GII-b  ARLESAEVNSMLTI..SEEALQLATISSFNG....DG..YNFTNVLGVSVYD.....PASGRVVQKRSFIEDLLFNKVVT
OH851-KJ399978-GII-c   ARLESVEVNSMLTI..SEEALQLATISSFNG....DG..YNFTNVLGVSVYD.....PASGRVVQKRSFIEDLLFNKVVT
GDS01-KM089829-GII-b   ARLESAEVNSMLTI..SEEALQLATISSFNG....DG..YNFTNVLGVSVYD.....PASGRVVQKRSFIEDLLFNKVVT
PDCoV-NH-KU981059      AQLDSREIINMFQT..SAQSLQLANITNFKG....D...YNFSSIL..............TTRLGGRSAIEDLLFNKVVT
SpDCoV_HKU17           ARLEAASIQSMLTY..NPQTVKLANITNFQS....DGIKYDLSSIL...........PVSTGS...RSAIEDLLFDKVVT
TGEV_Purdue_P115       ARLENMEVDSMLFV..SENALKLASVEAFNSSETLDPIYKEWPNIGGSWLEGLKYILPSHNSKRKYRSAIEDLLFDKVVT
SADS-CoV/GDGL01/2016   GILLDGDVSSLYSTIAAKTSPVVPTTDRFNV....SQ..FFLPKT............QSSANKYESRSAIEDLLFSKIET
                         965  970  975  980  985  990  995 1000 1005 1010 1015 1020 1025 1030 1035 1040
```

```
                       910       920       930       940       950       960       970       980
CV777-AF353511-GI-a    NGLGTVDEDYKRCSNGRSVADLVCAQYYSGVMVLPGVVDAEKLHMYSASLIGGMALGGITAAA.ALPFSYAVQARLNYLA
JS2008-KC210146-GI-b   NGLGTVDEDYKRCSNGRSVADLVCAQYYSGVMVLPGVVDAEKLHMYSASLIGGMALGGLTTAA.ALPFSHAVQARLNYLA
AH2012-KC210145-GII-a  NGLGTVDEDYKRCSNGRSVADLVCAQYYSGVMVLPGVVDAEKLHMYSASLIGGMVLGGFTSAA.ALPFSYAVQARLNYLA
AJ1102-JX188454-GII-b  NGLGTVDEDYKRCSNGRSVADLVCAQYYSGVMVLPGVVDAEKLHMYSASLIGGMVLGGFTAAA.ALPFSYAVQARLNYLA
OH851-KJ399978-GII-c   NGLGTVDEDYKRCSNGRSVADLVCAQYYSGVMVLPGVVDAEKLHMYSASLIGGMVLGGFTSAA.ALPFSYAVQARLNYLA
GDS01-KM089829-GII-b   NGLGTVDEDYKRCSNGRSVADLVCAQYYSGVMVLPGVVDAEKLHMYSASLIGGMVLGGFTAAA.ALPFSYAVQARLNYLA
PDCoV-NH-KU981059      SGLGTVDQDYKACSRDMAIADLVCSQYYNGIMVLPGVVDAEKMAMYTGSLTGAMVFGGLTAAA.AIPFATAVQARLNYVA
SpDCoV_HKU17           NGLGTVDQDYKKCTNGYSIADLVCAQYYNGIMVLPGVADPEKLAQYTASLTGAMVFGGLTSAA.AIPFSLAVQSRLNYVA
TGEV_Purdue_P115       SGLGTVDEDYKRCTGGYDIADLVCAQYYNGIMVLPGVANADKMTMYTASLAGGITLGALGGGAVAIPFAVAVQARLNYVA
SADS-CoV/GDGL01/2016   TGPGFYG.DYYNCKKN.AIQDLTCAQYHNGILVIPPIMDAETLGMYGGIAAASVTLGIFGGQAGMATWSVAMAGRLNALG
                         1045 1050 1055 1060 1065 1070 1075 1080 1085 1090 1095 1100 1105 1110 1115 1120
```

```
                       990       1000      1010      1020      1030      1040      1050      1060
CV777-AF353511-GI-a    LQTDVLQRNQQLLAESFNSAIGNITSAFESVKEAISQTSKGLNTVAHALTKVQEVVNSQGSALNQLTVQLQHNFQAISSS
JS2008-KC210146-GI-b   LQTDVLQRNQQLLAESFNSAIGNITSAFESVKEAISQTSNGLNTVAHALTKVQEVVNSQGSALTQLTIQLQHNFQAISSS
AH2012-KC210145-GII-a  LQTDVLQRNQQLLAESFNSAIGNITSAFESVKEAISQTSKGLNTVAHALTKVQEVVNSQGAALTQLTVQLQHNFQAISSS
AJ1102-JX188454-GII-b  LQTDVLQRNQQLLAESFNSAIGNITSAFESVKEAISQTSKGLNTVAHALTKVQEVVNSQGAALTQLTVQLQHNFQAISSS
OH851-KJ399978-GII-c   LQTDVLQRNQQLLAESFNSAIGNITSAFESVKEAISQTSKGLNTVAHALTKVQEVVNSQGAALTQLTVQLQHNFQAISSS
GDS01-KM089829-GII-b   LQTDVLQRNQQLLAESFNSAIGNITPAFESVKEAISQTSKGLNTVAHALTKVQEVVNSQGAALTQLTVQLQHNFQAISSS
PDCoV-NH-KU981059      LQTNVLQENQKILAESFNQAVGNISLALSSVNDAIQQTSEALNTVAIAIKKIQTVVNQQGEALSHLTAQLSNNFQAISTS
SpDCoV_HKU17           LQTDVLQRNQQILATSFNNAMGNITQAFHDVNQGLSQVAGAVTTIANAFTKIQDVVNAQGTALSTLTTQLTNNFQAISAS
TGEV_Purdue_P115       LQTDVLNKNQQILASAFNQAIGNITQSFGKVNDAIHQTSRGLATVAKALAKVQDVVNIQGQALSHLTVQLQNNFQAISSS
SADS-CoV/GDGL01/2016   VVQNALVDDVNKLANGFNQLTASV.............SKLALTTSSALQAIQAVVNQNAAQVESLVSGITENFGAISTN
                         1125 1130 1135 1140 1145 1150 1155 1160 1165 1170 1175 1180 1185 1190 1195 1200
```

```
                       1070      1080      1090      1100      1110      1120      1130      1140
CV777-AF353511-GI-a    IDDIYSRLDILSADVQVDRLITGRLSALNAFVAQTLTKYTEVQASRKLAQQKVNECVKSQSQRYGFCGGDGEHIFSLVQA
JS2008-KC210146-GI-b   IDDIYSRLDILSADVQVDRLITGRLSALNAFVAQTLTKYTEVQASRKLAQQKVNECVKSQSQRYGFCGGDGEHIFSLVQA
AH2012-KC210145-GII-a  IDDIYTRLDILSADVQVDRLITGRLSALNAFVAQTLTKYTEVQASRKLAQQKVNECVKSQSQRYGFCGGDGDHIFSLVQA
AJ1102-JX188454-GII-b  IDDIYSRLDILSADVQVDRLITGRLSALNAFVAQTLTKYTEVQASRKLAQQKVNECVKSQSQRYGFCGGDGEHIFSLVQA
OH851-KJ399978-GII-c   IDDIYSRLDILSADVQVDRLITGRLSALNAFVAQTLTKYTEVQASRKLAQQKVNECVKSQSQRYGFCGGDGEHIFSLVQA
GDS01-KM089829-GII-b   IDDIYSRLDILSADVQVDRLITGRLSALNAFVAQTLTKYTEVQASRKLAQQKVNECVKSQSQRYGFCGGDGEHIFSLVQA
PDCoV-NH-KU981059      IQDIYNRLEEVEANQQVDRLITGRLAALNAYVTQLLNQMSQIRQSRLLAQQKINECVKSQSSRYGFC.GNGTHIFSLTQT
SpDCoV_HKU17           IADIYNRLNQIEADAQVDRLITGRLAALNAFVTQTLAKLAEVRQSRQLALDKVNECVKSQSARYGFC.GNGTHLFSFSNA
TGEV_Purdue_P115       ISDIYNRLDELSADAQVDRLITGRLTALNAFVSQTLTRQAEVRASRQLAKDKVNECVRSQSQRFGFC.GNGTHLFSLANA
SADS-CoV/GDGL01/2016   FKVISQRLDKLEADVQMDRLINGRMNVLQLFVTNYKLKIAELRNTHRYVQSLINECVYAQSLRNGFC.GQGLHVLSLMQN
                         1205 1210 1215 1220 1225 1230 1235 1240 1245 1250 1255 1260 1265 1270 1275 1280
```

```
                       1150      1160      1170      1180      1190      1200      1210      1220
CV777-AF353511-GI-a    APQGLLFLHTVLVPGDFVNVLAIAGLCV...NGEIALTLREPGLVLFTHELQTYTATEYFVSSRRMFEPRKPTVSDFVQI
JS2008-KC210146-GI-b   APQGLLFLHTVLVPGDFVNVIAIDGLCV...NGDIALTLREPGLVLFTHELQNHTATEYFVSSRRMFEPRKPTVSDFVQI
AH2012-KC210145-GII-a  APQGLLFLHTVLVPGDFVDVIAIAGLCV...NDEIALTLREPGLVLFTHELQNHTATEYFVSSRRMFEPRKPTVSDFVQI
AJ1102-JX188454-GII-b  APQGLLFLHTVLVPGDFVNVIAIAGLCV...NDEIALTLREPGLVLFTHELQN.TATEYFVSSRRMYEPRKPTVGDFVQI
OH851-KJ399978-GII-c   APQGLLFLHTVLVPSDFVDVIAIAGLCV...NDEIALTLREPGLVLFTHELQNHTATEYFVSSRRMFEPRKPTVSDFVQI
GDS01-KM089829-GII-b   APQGLLFLHTVLVPGDFVNVIAIAGLCV...NDEIALTLREPGLVLFTHELQD.TATEYFVSSRRMYEPRKPTVGDFVQI
PDCoV-NH-KU981059      APNGIFFMHAVLVPNKFTRVNASAGICV...DNTRGYSLQ.PQLILYQ......FNNSWRVTPRNMYEPRLPRQADFIQL
SpDCoV_HKU17           APYGLMFFHTVLLPTQYATVQAYSGIC....HQNRALTLRDPSLALFQ......KDDKYLITPRNMYQPRTATKADFVYI
TGEV_Purdue_P115       APNGMIFFHTVLLPTAYETVTAWPGICASDGDRTFGLVVKDVQLTLFRN.....LDDKFYLTPRTMYQPRVATSSDFVQI
SADS-CoV/GDGL01/2016   APSGIMFFHYSLIPNNTITVKTTPGLCESDELGSKCIVAKDGVLVSA.......NLSYWQWSPRNLYKPENLTFANVIAV
                         1285 1290 1295 1300 1305 1310 1315 1320 1325 1330 1335 1340 1345 1350 1355 1360
```

```
                       1230      1240      1250      1260      1270      1280      1290
CV777-AF353511-GI-a    E.SCVVTYVNLTSDQLPDVIPDYIDVNKTLDEILASL.PNRTGPSLPLDVFNATYLNLTGEIADLEQRSESLRNTTEELR
JS2008-KC210146-GI-b   E.RCVVTYVNLTSDQLPDVIPDYIDVNKTLDEILASL.PNRIGPSLPLDVFNATYLNLTGEIADLEQRSESLRNTTEELR
AH2012-KC210145-GII-a  E.SCVVTYVNLTRDQLPDVIPDYIDVNKTLDEILASL.PNRTGPSLPLDVFNATYLNLTGEIANLEQRSESLRNTTEELQ
AJ1102-JX188454-GII-b  E.SCVVTYVNLTRDQLPDVIPDYIDVNKTLDEILASL.PNRTGPSLSLDVFNATYLNLTGEIADLEQRSESLRNTTEELQ
OH851-KJ399978-GII-c   E.SCVVTYVNLTRDQLPDLIPDYIDVNKTLDEILASL.PNRTGPSLPLDVFNATYLNLTGEIADLEQRSESLRNTTEELQ
GDS01-KM089829-GII-b   E.SCVVTYVNLTRDQLPEVIPDYIDVNKTLDEILASL.PNRTGPSLSLDVFNATYLNLTGEIADLEQRSESLRNTTEELQ
PDCoV-NH-KU981059      T.DCSVTFYNTTAANLPNIIPDVIDVNQTVSDIIDNL.PTATPPQWDVGIYNNTILNLTVEINDLQERSKNLSQIADRLQ
SpDCoV_HKU17           Q.SCDITFLNLTDTTIEAVIPDYVDVNKTIEEFLNNL.PNYTLPDLSIDRYNNTILNLTTEIADLNGKAANLSQIVEELE
TGEV_Purdue_P115       E.GCDVLFVNATVSDLPSIIPDYIDINQTVQDILENFRPNWTVPELTFDIFNATYLNLTGEIDDLEFRSEKLHNTTVELA
SADS-CoV/GDGL01/2016   SRGANYTTLNKTFD.IPELNSTF.PIEEEFREYFQNM......SSELQVLK....NLTADMSKLNISAE.....IQLIN
                         1365 1370 1375 1380 1385 1390 1395 1400 1405 1410 1415 1420 1425 1430 1435 1440
```

```
                             1300      1310      1320      1330      1340      1350      1360      1370
CV777-AF353511-GI-a          SLINNINNTLVDLEWLNRVETYIKWPWWVWLIIVIVLIFVVSLLVFCCISTGCC.GCCGCCGACFSGC..CRGPRLQPYE
JS2008-KC210146-GI-b         SLIYNINNTLVDLEWLNRVETYIKWPWWVWLIIFIVLIFVVSLLVFCCISTGCC.GCCGCCGACFSGC..CRGPRLQPYE
AH2012-KC210145-GII-a        SLIHNINNTLVDLEWLNRVETYIKWPWWVWLVIFIVLIFVVSLLVFCCISTGCC.GCCGCCCACFSGC..CRGPRLQPYE
AJ1102-JX188454-GII-b        SLIYNINNTLVDLEWLNRVETYIKWPWWVWLIIFIVLIFVVSLLVFCCISTGCC.GCCGCCGACFSGC..CRGPRLQPYE
OH851-KJ399978-GII-c         SLIYNINNTLVDLEWLNRVETYIKWPWWVWLIIFIVLIFVVSLLVFCCISTGCC.GCCGCCCACFSGC..CRGPRLQPYE
GDS01-KM089829-GII-b         SLIYNINNTLVDLEWLNRVETYIKWPWWVWLIIFIVLIFVVSLLVFCCISTGCC.GCCGCCGACFSGC..CRGPRLQPYE
PDCoV-NH-KU981059            NYIDNLNNTLVDLEWLNRVETYLKWPWYIWLAIALALIAFVTILITIFLCTGCCGGCFGCCGGCFGLF..SKKKRYTDDQ
SpDCoV_HKU17                 QYIKNINSTLVDLEWLNRVETYIKWPWYIWLAIALAFTAFVAILITIFLCTGCCGGCFGCCGGCFGLF..SKKKRYTDDH
TGEV_Purdue_P115             ILIDNINNTLVNLEWLNRIETYVKWPWYVWLLIGLVVIFCIPLLLFCCCSTGCC.GCIGCLGSCCHSI..CSRRQFENYE
SADS-CoV/GDGL01/2016         EIAHNVSNMRVEVEKFQRYVNYVKWAWWQWLLIFIALTLLAGLMLWCCLATGCC.GMCGCLAATCASCCDCRGTKLQSYE
                               1445 1450 1455 1460 1465 1470 1475 1480 1485 1490 1495 1500 1505 1510 1515 1520

                             1380
CV777-AF353511-GI-a          AFEKVHVQX..
JS2008-KC210146-GI-b         AFEKVHVQX..
AH2012-KC210145-GII-a        VFEKVHVQX..
AJ1102-JX188454-GII-b        AFEKVHVQX..
OH851-KJ399978-GII-c         VFEKVHVQX..
GDS01-KM089829-GII-b         AFEKVHVQX..
PDCoV-NH-KU981059            PTPSFKFKEWX
SpDCoV_HKU17                 PTPSFKFKEWX
TGEV_Purdue_P115             PIEKVHVHX..
SADS-CoV/GDGL01/2016         .IEKVHVQX..
                               1525 1530
```

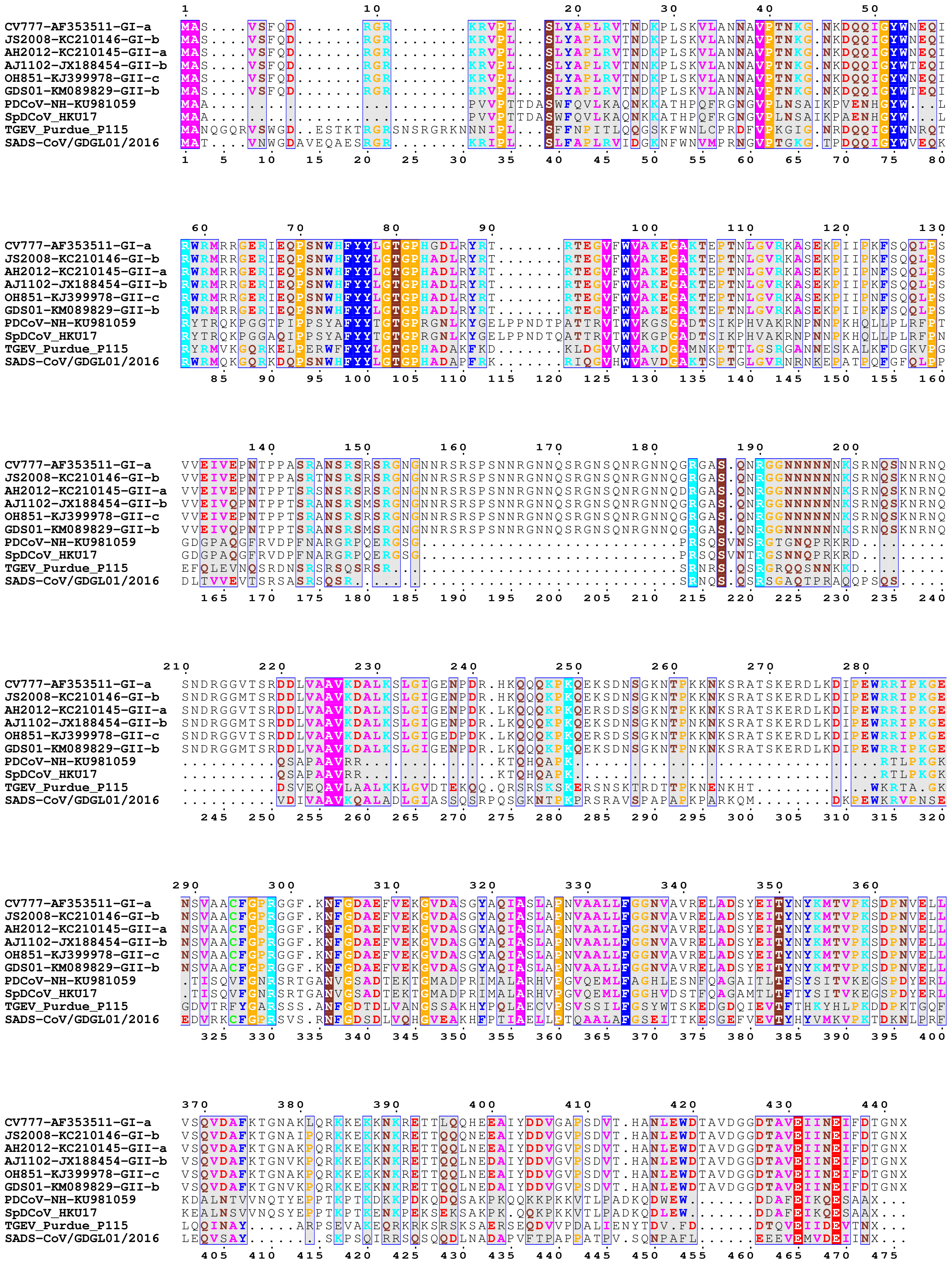
CV777-AF353511-GI-a
JS2008-KC210146-GI-b
AH2012-KC210145-GII-a
AJ1102-JX188454-GII-b
OH851-KJ399978-GII-c
GDS01-KM089829-GII-b
PDCoV-NH-KU981059
SpDCoV_HKU17
TGEV_Purdue_P115
SADS-CoV/GDGL01/2016
