# Supplementary material for: Molecular and Structural Evolution of Porcine Epidemic Diarrhea Virus
Source: Animals (Basel). 2022 Dec 1;12(23):3388. doi: 10.3390/ani12233388 (PMC9736354; doi:10.3390/ani12233388)
Supplement: Supplementary file 1 [file animals-12-03388-s001.zip › Supplementary Table S2.pdf]

Supplementary Table S2. Codon usage bias of the 59 sense codons of PEDV CV777.

Codon	Amino acid	RSCU	Preference
GCT	A	1.88	pref
GCC	A	0.68	unpref
GCA	A	1.12	pref
GCG	A	0.28	unpref
TGT	C	1.34	pref
TGC	C	0.66	unpref
GAT	D	1.28	pref
GAC	D	0.72	unpref
GAA	E	0.92	unpref
GAG	E	1.08	pref
TTT	F	1.34	pref
TTC	F	0.66	unpref
GGT	G	2.36	pref
GGC	G	1	pref
GGA	G	0.4	unpref
GGG	G	0.2	unpref
CAT	H	1.36	pref
CAC	H	0.64	unpref
ATT	I	2.4	pref
ATC	I	0.84	unpref
ATA	I	0.76	unpref
AAA	K	0.7	unpref
AAG	K	1.3	pref
TTA	L	0.78	unpref
TTG	L	1.62	pref
CTT	L	1.92	pref
CTC	L	0.66	unpref
CTA	L	0.48	unpref
CTG	L	0.6	unpref
AAT	N	1.28	pref
AAC	N	0.72	unpref
CCT	P	1.92	pref
CCC	P	0.56	unpref
CCA	P	1.32	pref
CCG	P	0.2	unpref
CAA	Q	0.88	unpref
CAG	Q	1.12	pref
CGT	R	2.22	pref
CGC	R	1.14	pref
CGA	R	0.36	unpref
CGG	R	0.24	unpref
AGA	R	1.14	pref
AGG	R	0.9	unpref
TCT	S	1.86	pref
TCC	S	0.72	unpref
TCA	S	1.02	pref
TCG	S	0.24	unpref
AGT	S	1.38	pref
AGC	S	0.66	unpref
ACT	T	1.84	pref
ACC	T	0.68	unpref
ACA	T	1.16	pref
ACG	T	0.32	unpref
GTT	V	2	pref
GTC	V	0.72	unpref
GTA	V	0.48	unpref
GTG	V	0.76	unpref
TAT	Y	1.34	pref
TAC	Y	0.66	unpref

The RSCU values of the 59 sense codons (excluding ATG and TGG) were calculated. The codons with RSCU > 1 were defined as preferred (pref) codons and those with RSCU < 1 were defined as unpreferred (unpref) codons.
