# Supplementary figures and images for: Molecular and Structural Evolution of Porcine Epidemic Diarrhea Virus
Source: Animals (Basel). 2022 Dec 1;12(23):3388. doi: 10.3390/ani12233388 (PMC9736354; doi:10.3390/ani12233388)

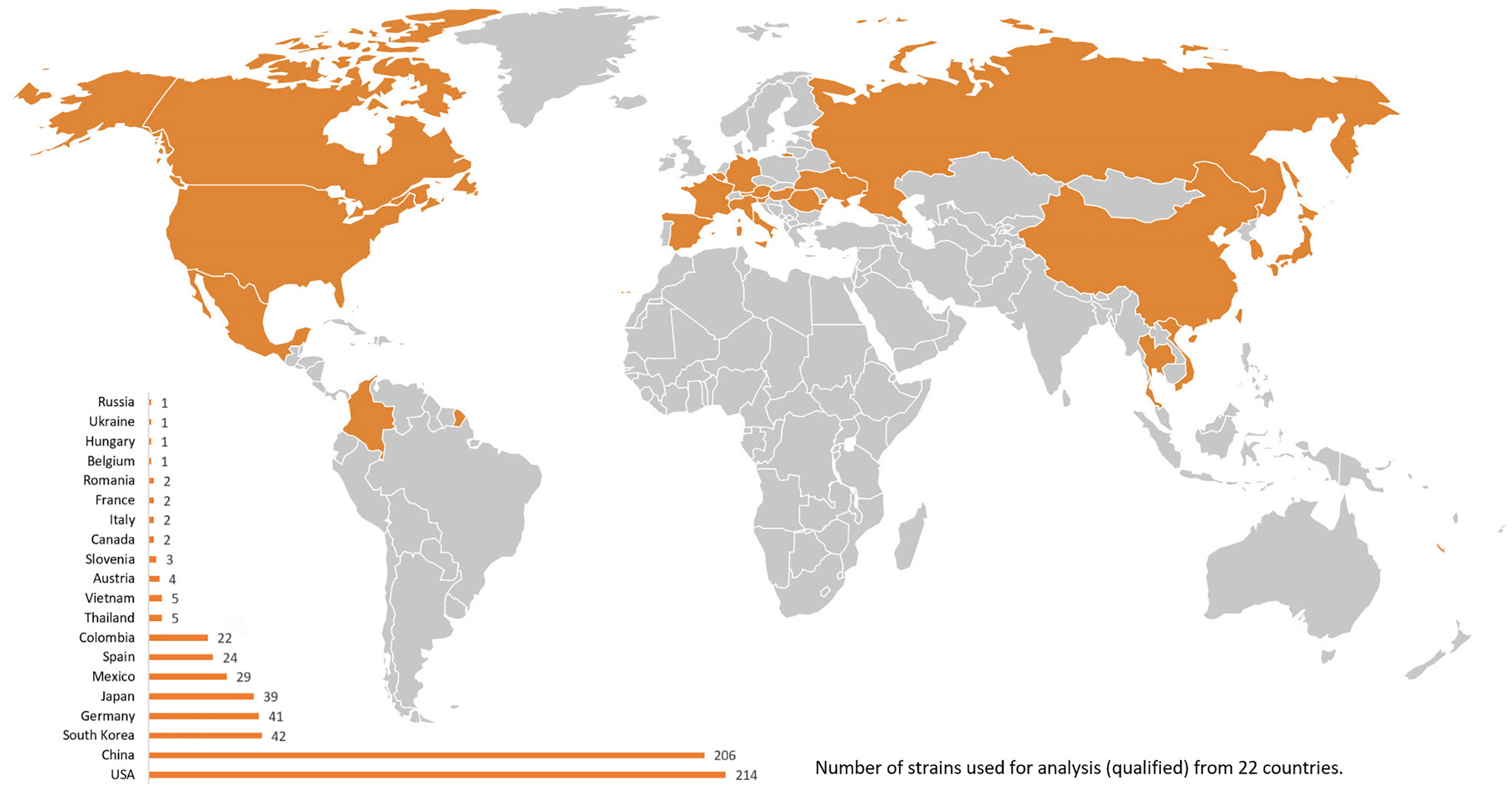

Supplement: Supplementary file 1 [file animals-12-03388-s001.zip › Supplementary Figure S1 location.jpg]

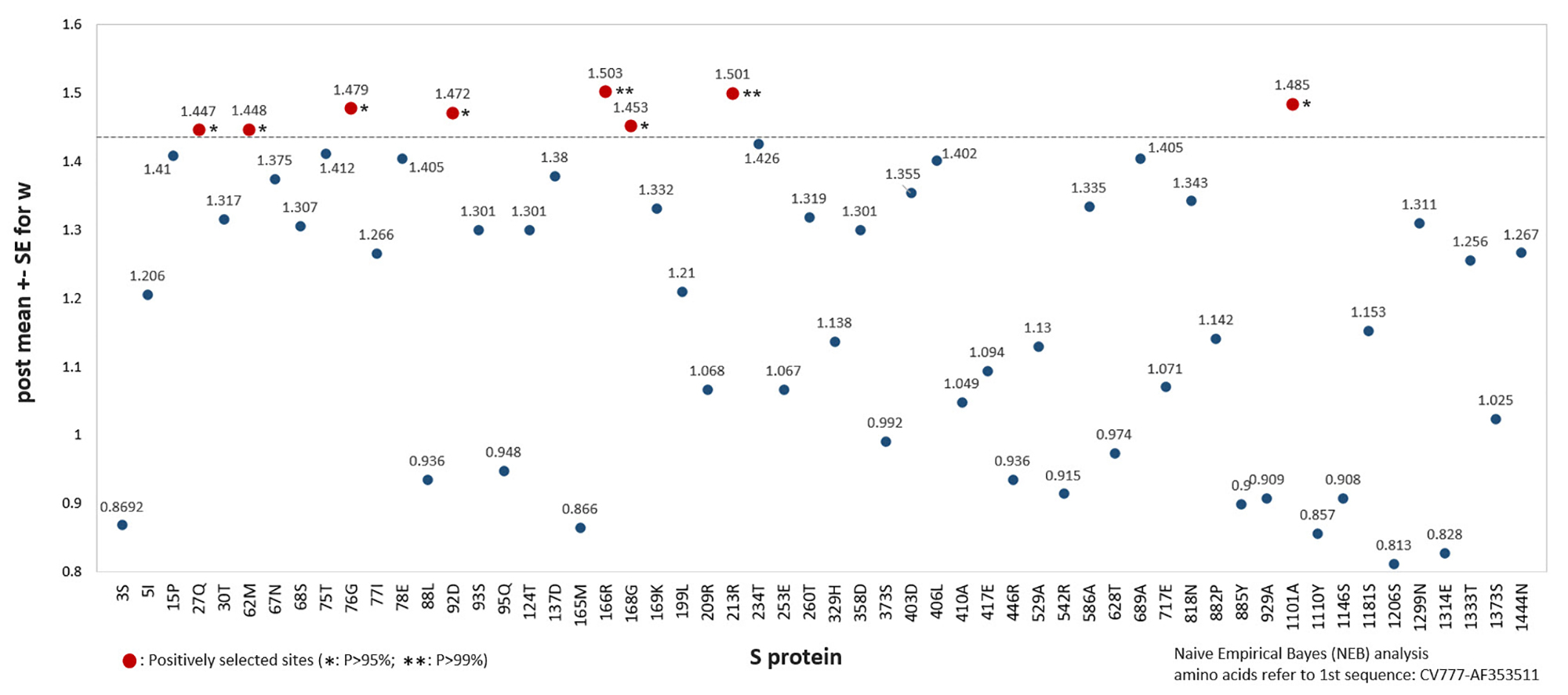

Supplement: Supplementary file 1 [file animals-12-03388-s001.zip › Supplementary Figure S2 positive selection.jpg]
